# Enhanced Hydrogen Generation through Low-Temperature Plasma Treatment of Waste Aluminum for Hydrolysis Reaction

**DOI:** 10.3390/ma17112637

**Published:** 2024-05-29

**Authors:** Marius Urbonavicius, Sarunas Varnagiris, Ainars Knoks, Ansis Mezulis, Janis Kleperis, Christiaan Richter, Rauan Meirbekova, Gudmundur Gunnarsson, Darius Milcius

**Affiliations:** 1 Center for Hydrogen Energy Technologies, Lithuanian Energy Institute, 3 Breslaujos, 44403 Kaunas, Lithuania; marius.urbonavicius@lei.lt (M.U.); sarunas.varnagiris@lei.lt (S.V.); 2 Institute of Solid State Physics, University of Latvia, LV-1063 Riga, Latvia; ainars.knoks@cfi.lu.lv (A.K.); ansis.mezulis@cfi.lu.lv (A.M.); janis.kleperis@cfi.lu.lv (J.K.); 3 Faculty of Industrial Engineering, Mechanical Engineering and Computer Science, University of Iceland, 102 Reykjavik, Iceland; cpr@hi.is; 4 IceTec, 112 Reykjavik, Iceland; rauan@taeknisetur.is (R.M.); gudmundur@taeknisetur.is (G.G.)

**Keywords:** waste aluminum, plasma treatment, recycling, waste reduction, hydrogen generation, hydrolysis, electricity generation, PEM fuel cell

## Abstract

This study investigates the low-temperature hydrogen plasma treatment approach for the improvement of hydrogen generation through waste aluminum (Al) reactions with water and electricity generation via proton-exchange membrane fuel cell (PEM FC). Waste Al scraps were subjected to ball milling and treated using two different low-temperature plasma regimes: Diode and magnetron-initiated plasma treatment. Hydrolysis experiments were conducted using powders with different treatments, varying molarities, and reaction temperatures to assess hydrogen generation, reaction kinetics, and activation energy. The results indicate that magnetron-initiated plasma treatment significantly enhances the hydrolysis reaction kinetics compared to untreated powders or those treated with diode-generated plasma. Analysis of chemical bonds revealed that magnetron-initiated hydrogen plasma treatment takes advantage by promoting a dual procedure: Surface cleaning and Al nanocluster deposition on top of Al powders. Moreover, it was modeled that such H_2_ plasma could penetrate up to 150 Å depth. Meanwhile, electricity generation tests demonstrate that only 0.2 g of treated Al powder can generate approximately 1 V for over 300 s under a constant 2.5 Ω load and 1.5 V for 2700 s with a spinning fan.

## 1. Introduction

Hydrogen has emerged as a crucial energy carrier in sustainable energy solutions’ global aim due to its unique properties and versatile applications. Hydrogen offers an exceptional potential for clean energy production and storage. Its combustion generates only water as a by-product, making it an environmentally friendly alternative to fossil fuels, particularly in mitigating greenhouse gas emissions and combating climate change. Furthermore, hydrogen exhibits high energy density, enabling its utilization across various sectors, including transportation, industry, and power generation [1,2,3].

Despite the huge effort to shift the hydrogen economy to the clean energy sector, the majority of hydrogen is still produced via steam methane reforming (SMR), which stands as the predominant industrial process, utilizing high-temperature steam to react with methane, yielding hydrogen and substantial amounts of carbon-based emissions. Therefore, the growing interest in alternative hydrogen production methods is emphasized due to the transition towards sustainable energy systems, including electrolysis, solar water splitting, biomass gasification, thermochemical water splitting, or hydrolysis reaction [4,5,6,7,8]. While these methods have their own advantages and disadvantages, the hydrolysis reaction stands out from others due to unique characteristics, such as: (i) the process avoids external high temperatures or electricity provision; (ii) the hydrogen can be produced on-board; (iii) due to the exothermic reaction origin, it generates heat, which can be used as an additional reaction outcome; (iv) the reaction requires a reductant and water only; and (v) there are no carbon-based emissions during the reaction [9,10,11].

Since there are considerable research attempts and notable advancements using various metal-based materials for hydrolysis reactions, the majority of researchers focus on magnesium-based and aluminum-based compounds [12,13,14,15,16,17,18,19]. Furthermore, the current global emphasis on sustainability and environmentally friendly solutions motivates scientists to explore the utilization of waste materials as potential energy sources, in line with the principles of the circular economy. According to estimates, the aluminum market has experienced notable growth and is projected to reach 38 million tons by 2025, with consistent 4–5% annual growth rates in recent years [20]. Although recycled aluminum has seen growth, these figures directly contribute to the rising quantity of waste aluminum in industrial processes such as manufacturing window frames, automotive production, and other sectors, as indicated by the growing number of alloys in use and the lack of a clear recycling pathway [21].

Aluminum is one of the most abundant metals in the Earth’s crust, constituting approximately 8.1% of its composition. It is widely available, cost-effective, lightweight, and boasts a high electron density. During the reaction between aluminum and water, up to 1.245 liters of hydrogen gas per gram of aluminum metal can be produced under standard conditions. The reaction by-product Al(OH)_3_ can be converted into valuable material such as Al_2_O_3_, which can be used in catalysts or other industries. Moreover, the produced hydrogen is moist and warm, rendering it ideal for supplying low-temperature fuel cells [13,16,19]. Therefore, considering process sustainability, the properties of aluminum, the significant amount of hydrogen generated, and the valuable reaction by-products, utilizing industrial waste aluminum for hydrogen production through water reaction appears as a promising solution for practical implementation.

However, a notable obstacle hindering the practical application of the aluminum hydrolysis process is the inert aluminum oxide layer that naturally forms on the surface of pure aluminum, impeding the hydrogen generation process. Various techniques have been examined to remove the passive oxide, including mechanical activation of Al [22,23,24], liquid metal activation and alloying [25,26,27], activation of Al in alkaline or acidic solutions [28,29,30], activation by Al_2_O_3_ or Al(OH)_3_ [31,32,33], activation with carbon-based materials [34,35,36], and others [37,38,39,40]. Significant improvements were achieved using these methods, mostly related to aluminum–water reaction kinetics. For instance, F. Q. Wang et al. analyzed the effects of low-melting-point metals (Ga, In, and Sn) on the hydrolysis properties of aluminum alloys. They enhanced the yield of H_2_ by alloying Al–In–Sn with a mass ratio of In to Sn at 1:4, speculating that the introduction of Ga elements facilitates the creation of defects within the Al alloys and promotes the formation of Ga–In3Sn–InSn4 eutectic alloys on the surface of the alloys [41]. Another group led by F. D. Manilevich examined mechanochemically activated aluminum powders made of Ga–In–Sn or Ga–In–Sn–Zn eutectic alloys (5 wt.%) and graphite (1–3 wt.%) in a mixer-type ball mill for generating hydrogen from water. They enhanced the hydrogen evolution rate value by more than an order of magnitude using pellets containing graphite additives compared to those without graphite [42]. The hydrogen generation kinetics were improved by treating Al with a series of strong acid or alkaline solutions, including HCl, NaOH, NaAlO_2_, and a mixture of NaAlO_2_ + Al(OH)_3_, by N. Wang et. al. Such an approach leads to an increase in hydrogen generation rate of about 7 times, from ~5 mL min^−1^ to 37 mL min^−1^ [43].

Overall, these methods typically incorporate various additives to activate aluminum or modify the solution’s molarity, which could destroy the aluminum oxide layer. Despite a significant increase in hydrogen generation rate in these methods, such additives can introduce contaminants to the reaction by-product, limiting its potential further application or potentially compromising the purity of the produced hydrogen gas, particularly in high-temperature hydrolysis reactions.

As an alternative to the previously listed activation methods, magnetron generated low-temperature plasma treatment can be used as a viable solution. Low-temperature plasma treatment offers a versatile and efficient way to adjust the surface properties of various materials, enabling diverse applications. This technique is environmentally friendly as it avoids the use of additional chemicals or additives that could potentially harm our environment. This process involves subjecting materials to a low-temperature plasma environment at reduced gas pressures. During a sequence of ionization and excitation events, the plasma generates reactive species such as ions, radicals, and electrons, initiating interactions with the treated material surface. This, in turn, encourages the removal of surface contaminants through physical and chemical interactions and/or creates microstructures like cracks or pores according to selected plasma treatment parameters [44,45,46,47].

There are a number of works showing the successful application of such a method in various different fields [48,49,50,51,52,53]. For instance, the group led by Magno B. Costa showed that plasma treatment could improve the semiconductor Sb_2_Se_3_ characteristics for solar-driven hydrogen generation via water splitting. They disclosed that N_2_ could increase hydrophilic characteristics by creating Sb–N bonds and, in turn, improve wettability during the reaction [54]. Another group employed Ar plasma treatment on few-layer MoS_2_ thin films for enhanced photocatalytic properties 1.4 times by hydrogen production by water splitting. They involved defect engineering, indicating that Ar plasma treatment creates defects in the form of sulphur vacancies, while decomposition of the sulfur was done from pristine MoS_2_ [55]. Meanwhile, hydrogen plasma treatment on the NiFe/CeO_2_ catalyst was tested for further CO_2_ methanation procedures. The scientists revealed that plasma treatment increased the CO_2_ conversion and CH_4_ selectivity of the NiFe/CeO_2_ catalysts, resulting in stable activity for 150 h on the stream [56].

Nevertheless, despite the considerable interest in aluminum activation techniques for generating hydrogen through hydrolysis reactions, there have been almost no efforts to implement plasma treatment. In our previous work, we employed a low-temperature glow discharge plasma activation approach for pure aluminum powder. The results showed a significant increment in hydrogen generation kinetics and the aluminum surface shift from non-polar to polar chemically bound groups, enhancing surface hydrophilicity [57].

In this work, we focus on different types of low-temperature plasma treatment utilizing diode and magnetron-initiated plasma treatment techniques. The initiation of magnetron-generated plasma could yield a denser structure, generating more reactive species compared to diode generated plasma. Consequently, it may induce a more pronounced formation of surface defects on aluminum powder. To our knowledge, such a plasma treatment approach for aluminum activation has never been tested. In addition, we utilized waste aluminum scraps sourced from the window frame industry as our primary material. The results revealed that such a technique could be successfully applied for aluminum powder alteration and, in turn, increase hydrogen generation kinetics through the hydrolysis process.

## 2. Materials and Methods

### 2.1. Ball Milling

**Figure 1:**
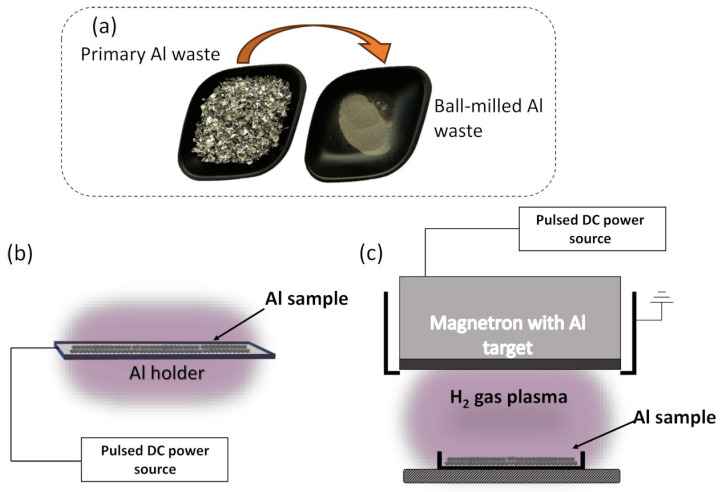
Al waste materials and principal treatment scheme: (**a**) primary Al waste and ball-milled Al waste as powder, (**b**) diode low-temperature plasma treatment scheme, and (**c**) magnetron-initiated low-temperature plasma treatment scheme.

Aluminum waste materials (Figure 1a) were obtained from the company “Stiklita, JSC”. Stiklita specializes in manufacturing aluminum frames for insulating glass units and various household applications [58]. In order to have better surface proximity and material immersion in plasma during the second step, the aluminum scraps were ball milled using planetary ball milling (Pulverisette 6, Weimar, Germany). Stainless steel balls, each with a diameter of 10 mm and a mass of 4 g, were utilized, keeping the balls to powder weight ratio at 1:10. Before the procedure, the system was pumped using a rotary vacuum pump to avoid powder surface contamination with air molecules. The total ball-milling time was selected at 8 h with 96 cycles (one cycle consisted of 5 min ball-milling and 2 min pause). Ball-milling speed was set at 200 rpm. Al waste powder after the ball-milling procedure is presented in Figure 1a.

### 2.2. Low Temperature Plasma Treatment

After the ball-milling procedure, the Al powder was treated under low-temperature plasma using two different regimes. In the case of the diode low-temperature plasma treatment (Figure 1b), the plasma was initiated using an Al holder, meaning that Al powder was fully immersed into the plasma. The voltage during the plasma treatment was 500 V (P = 350 W). The second regime-magnetron-initiated low-temperature plasma treatment (Figure 1c) includes Al magnetron for plasma generation, while the Al powder holder was kept at a distance of 5 cm from the magnetron. The voltage during the plasma treatment was 200 V (P = 150 W). In both cases, treatments were performed using H_2_ as a working gas with a working pressure of 1 × 10^−1^ mbar. In both cases, a 3 h treatment time was selected. These parameters were selected based on our previous experience, which showed optimal results under these conditions. The pressure was chosen to achieve the maximum hydrogen amount inside the chamber while maintaining a stable plasma treatment regime. The voltages were selected to ensure consistent operation throughout the treatment.

### 2.3. Characterization

The ball-milled powder morphology was analyzed by a scanning electron microscope (SEM, Hitachi S-3400 N, Tokyo, Japan) with a 5 kV accelerating voltage before and after plasma treatment using a secondary electron detector. The surface elemental composition and chemical bound analysis were performed using an X-ray photoelectron spectrometer (XPS, PHI Versaprobe 5000, Boston, MA, USA). The main XPS measurement parameters were monochromated 1486.6 eV Al radiation, 25 W beam power, 100 μm beam size, and a 45° measurement angle. Charging of the samples was offset by employing a dual neutralization system, which comprised a low-energy electron beam and an ion beam. Also, all obtained spectra were shifted by setting the adventitious carbon C1s peak at 284.8 eV before the analysis.

The X-ray diffractometer (XRD, Bruker D8, Hamburg, Germany) was utilized to examine the crystal structure of both primary and plasma-treated Al powder. Measurements were conducted within the 2Θ range of 20–70°, employing Cu Kα radiation (λ = 0.15406 nm) and a Lynx Eye linear position-sensitive detector.

The penetration of low-temperature hydrogen-plasma ions into the aluminum powder, generated by the magnetron, was assessed using SRIM (version SRIM-2008.04), a freely available Monte Carlo simulation code known as Stopping and Range of Ions in Materials. SRIM is widely used for sputtering calculations and related applications. It is built upon the TRIM code (Transport of Ions in Matter), incorporating Biersack’s magic formula and the ZBL (Ziegler–Biersack–Littmark) universal interaction potential for accurate calculations.

### 2.4. Hydrogen Generation

The hydrogen generation experiments via aluminum reaction with water were initiated using a custom-made laboratory stand. The reaction flask was immersed in a water bath with the possibility of controlling the temperature. During the hydrolysis reaction, 0.2 g of aluminum powder and 50 mL of water alkali solution (0.5 M) were used. The generated hydrogen gas was first transferred to an interim flask with cold water. The flask served as a condensing vessel to eliminate water moisture from the H_2_ product stream before measuring the H_2_ content. Then H_2_ gas reached the inverted burette, where the quantification of hydrogen production yield was evaluated by the decrease in water level in the burette. The rate of the H_2_ generation reaction was determined by integrating the flow of H_2_ over time.

### 2.5. Fuel Cell Electricity Generation

The experiments on electricity generation with a fuel cell were conducted following a similar procedure as for the hydrolysis reaction. However, instead of using an inverted burette, the produced H_2_ was directed to the 1 W PEM fuel cell (Horizon Fuel Cell Europe, Prague, Czech Republic). The Horizon renewable energy monitor (software version 1.3) was employed to record and display the voltage generated during the reaction. Two regimes were tested for electricity generation: a constant resistance of 2.5 Ω and a spinning fan. Both were applied as external loads.

## 3. Results and Discussion

### 3.1. Hydrogen Production

**Figure 2:**
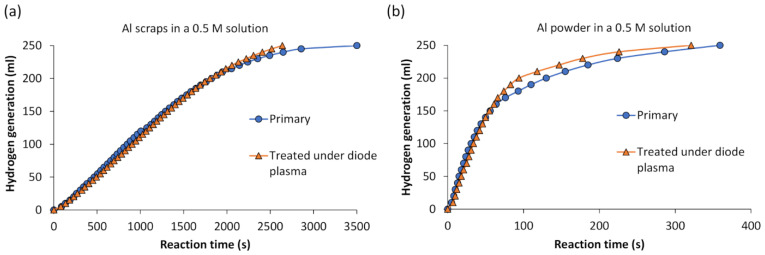
Experimentally measured cumulative hydrogen production from (**a**) primary Al scraps and diode plasma-treated scraps, (**b**) primary Al powder and diode-plasma-treated powder.

Firstly, experiments were conducted to produce hydrogen, comparing primary aluminum with aluminum treated under diode plasma. These experiments utilized both aluminum scraps (Figure 2a) and aluminum powders (Figure 2b) immersed in alkali solution to promote the reaction with water (0.2 g of Al in each experiment). No distinct trends are observed in hydrogen production between untreated primary aluminum scraps and diode-plasma-treated scraps in the presence of a 0.5 M NaOH alkali solution (Figure 2a). While both sets of scraps demonstrated a steady increase in hydrogen generation over time and the curves show considerable overlap, the diode-plasma-treated scraps exhibit a slightly accelerated reaction rate at the end, reaching a maximum of 250 mL in 2639 s compared to 3500 s for primary scraps (Figure 2a).

Moreover, the aluminum powder obtained after ball milling of primary aluminum scraps demonstrated enhanced reactivity, achieving the maximum hydrogen yield of 250 mL in a shorter time frame of 359 s (Figure 2b). Combining ball milling with diode-plasma treatment yielded slightly improved results, as observed in the diode-plasma-treated ball-milled scraps, with a peak hydrogen yield of 250 mL reached in 321 s.

The data suggests that combining ball milling and diode-plasma treatment has a slight synergistic effect on hydrogen production from aluminum scraps. It is apparent that ball milling increases the surface area of the aluminum, enhancing reactivity, while diode-plasma treatment shows a possibility to further enhance this reactivity by potentially modifying the surface chemistry of the aluminum. However, despite the slight enhancement, the improvements in reaction rates with diode-plasma treatment are not substantial compared to untreated primary aluminum or ball-milled scraps.

The goal is to enhance reactivity further. Therefore, considering the promising results seen with diode-plasma treatment, the logical next step is to explore alternative plasma treatment methods that may offer greater improvements. Magnetron plasma treatment presents itself as a viable option, offering potentially higher energy densities and different plasma characteristics that could lead to more significant enhancements in hydrogen production efficiency.

Recognizing that diode-plasma treatment alone may not maximize the reactivity of the aluminum material, attention was directed towards investigating the potential of magnetron plasma to bring more significant alterations to the surface properties of both aluminum scraps and powder. Magnetron plasma treatment offers numerous advantages over diode-plasma treatment, especially concerning the alteration of material surface properties. Magnetron plasma systems typically operate at higher energy densities than diode-plasma systems, allowing for more efficient activation of surface species and deeper material penetration. This capability can potentially lead to more substantial modifications and improvements in reactivity. Furthermore, magnetron plasma systems can achieve higher levels of ionization efficiency compared to diode-plasma systems, resulting in a greater abundance of reactive species such as ions and radicals. These species can interact more effectively with the surface of aluminum scraps, facilitating desired chemical reactions and surface modifications.

**Figure 3:**
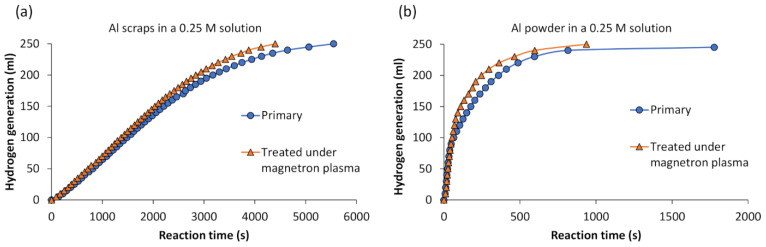
Experimentally measured cumulative hydrogen production from (**a**) primary and magnetron plasma-treated Al scraps, (**b**) primary and magnetron plasma-treated Al powder.

Additionally, adjustments were made to decrease the alkali concentration (down to 0.25 M) in the reaction environment, aiming to isolate the impact of plasma treatment from other chemical factors. This reduction aimed to isolate the effects of plasma treatment from other chemical factors, thereby enhancing the understanding of the specific impact of plasma treatment on aluminum, independent of other variables. Curves illustrating the cumulative hydrogen production using 0.2 g of primary and magnetron plasma-treated Al scraps and powder are depicted in Figure 3. In this instance, it is evident that magnetron plasma treatment has a more pronounced positive effect on both aluminum scraps and powder (Figure 3a,b). We can observe that magnetron plasma-treated Al scraps exhibited a difference in hydrogen production during the latter part of the reaction, increasing from 150 mL of H_2_ reached in 2033 s to 250 mL in 4398 s, compared to 2210 s and 5547 s for primary aluminum scraps, respectively. The most significant effect was observed with aluminum powder treated under magnetron plasma, where after approximately 54 s, the curve steepened, progressing from the point of producing 100 mL of H_2_ to reaching 250 mL of H_2_ in 937 s, compared to 1778 s for primary aluminum powder.

These findings underscore the effectiveness of magnetron plasma treatment, particularly in enhancing the reactivity of aluminum powder for hydrogen production. It is presumed that the greater surface area of aluminum powder enables more efficient interaction with the plasma, resulting in the observed enhanced reactivity and faster hydrogen production kinetics.

**Figure 4:**
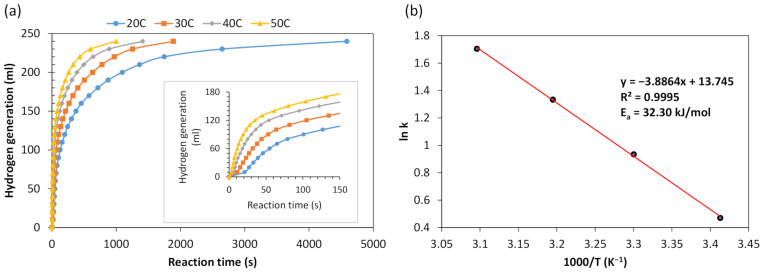
(**a**) Impact of temperature on hydrogen production kinetics using magnetron plasma-treated Al powder, (**b**) Arrhenius regression plot of Al powder.

Additionally, reactions between Al powder treated under magnetron plasma and an alkali solution of 0.25 M were performed at different temperatures (20 °C, 30 °C, 40 °C, and 50 °C), and the results are presented in Figure 4a.

To assess the reaction kinetics of powdered aluminum scraps, the activation energy was determined using the Arrhenius equation [59]:

ln *k* = ln *A* − *E_a_*/*RT*, (1)

Here, *k* is the reaction rate constant (ml·s^−1^), *E_a_* is the activation energy of the reaction (J·mol^−1^), *A* is the pre-exponential factor, *R* is the gas constant (8.314 J·mol^−1^·K^−1^), and *T* is the temperature of the solution (K). The Arrhenius plot was generated by focusing on the initial stage of the reaction, which exhibited a nearly linear dependence of the generated H_2_ volume on time, as depicted in the insert of Figure 4a. The slopes of these graphic lines represent the rate of produced H_2_, consistent with zero-order hydrolysis. Additionally, according to M. Huang et al., if the R^2^ value exceeds 0.99 (0.9995 in our case), then the Arrhenius equation adequately describes the hydrolysis process [60]. Figure 4b illustrates the Arrhenius plot. The activation energy for the hydrolysis reaction of powdered aluminum scraps was determined to be 32.30 kJ·mol^−1^. A lower activation energy indicates higher performance in hydrogen generation from the solution, and vice versa.

It is noteworthy that the activation energy of aluminum powder treated under magnetron plasma falls within the range or, in some instances, even lower than that reported by other researchers investigating hydrolysis reactions with aluminum. The studies have documented activation energies ranging from 22.57 to 73.0 kJ mol^−1^ [59,61,62,63]. The lowest activation energy was attained through ball milling Al–Bi–Li mixtures with NaCl, while the highest activation energy was observed with Al powder promoted by sodium stannate [62,63].

Furthermore, it was decided to reduce the amount of NaOH dissolved in pure water even further. This adjustment aimed to more accurately discern the impact of plasma as opposed to the alkaline solution. Additionally, for practical applications involving electricity generation, it is advisable to utilize pure water or implement a filtering system between the fuel cell and hydrogen generator. The presence of alkaline species, accompanying the produced hydrogen, could potentially damage the fuel cell membrane, particularly in the case of the PEM fuel cell, which is highly sensitive to impurities.

**Figure 5:**
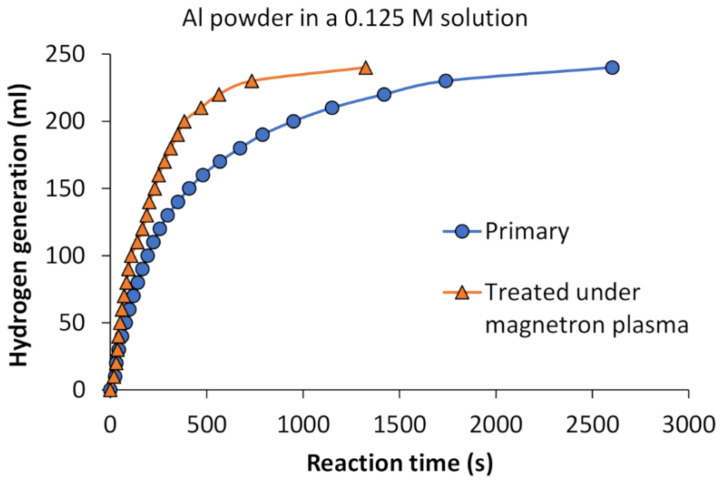
Experimentally measured cumulative hydrogen production from primary Al and magnetron plasma-treated Al powder.

Hence, the subsequent hydrolysis experiment was conducted using the same quantity of powdered aluminum but with a reduced alkaline solution concentration (0.125 M). As depicted in Figure 5, aluminum powder treated under magnetron plasma exhibited a superior hydrogen generation rate compared to primary aluminum powder under identical reaction conditions. Although both reactions commenced almost simultaneously, the hydrogen generation curve of the magnetron plasma treated aluminum powder exhibited a more pronounced ascent. To produce the same amount of hydrogen (240 mL), it took 1325 s for plasma-treated aluminum and 2603 s for primary aluminum powder. The influence of plasma appears to offer a promising approach for enhancing the reaction rate, even at lower molarities. Plasma treatment holds promise for advancing sustainable hydrogen production from aluminum scraps, offering implications for clean energy technologies and waste utilization. However, further explorations into the underlying mechanisms and optimization strategies are essential to fully harnessing the potential of this method.

### 3.2. Surface Chemical Analysis by XPS Technique

**Figure 6:**
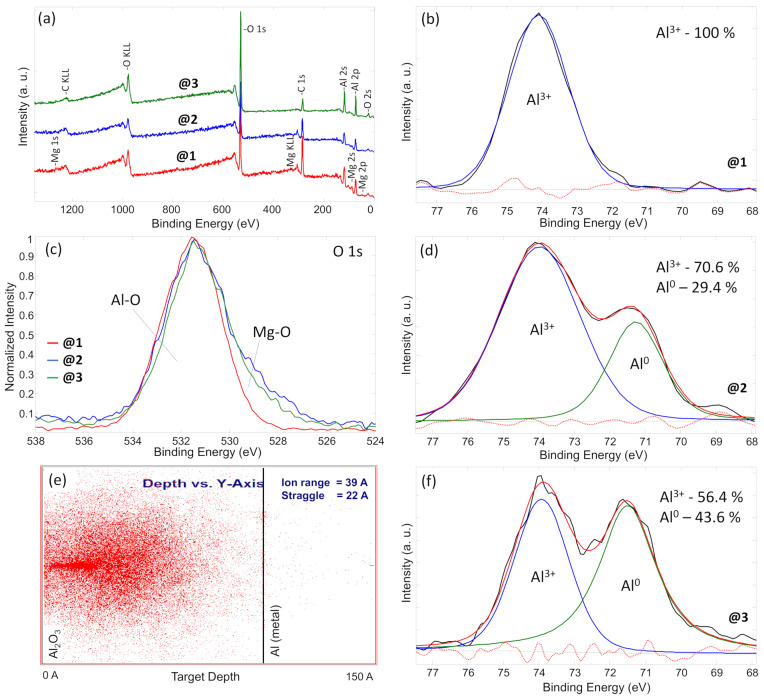
XPS results: (**a**) Survey spectra and (**c**) O 1s spectra of @1, @2 and @3; (**f**) Al 2p spectra of ball-milled Al powder, (**d**) treated under magnetron plasma Al powder and (**b**) synthesized Al nanoparticles; (**e**) modelling of H_2_ plasma penetration depth. “@1” represents information and spectra of ball-milled Al powder, “@2”—treated under magnetron plasma Al powder and “@3”—synthesized Al nanoparticles. Black lines represent measurement curves and dashed red lines—background.

The XPS analysis is an appropriate way to understand the plasma process influence on the target top layer alteration due to its up to 10 nm analysis range. For this reason, surface elemental composition and chemical bond analysis were performed and presented in Table 1 and Figure 6, respectively. In addition, a separate test was conducted using a quartz substrate, which was treated under magnetron-initiated plasma using the same conditions as during Al powder treatment. This was performed to determine whether magnetron sputtering induces aluminum deposition from the cathode, despite the use of hydrogen plasma, which primarily consists of light ions that are not prone to sputtering the magnetron target and leading to film deposition. Following the magnetron plasma treatment, the quartz substrate remained as transparent as it was before the test. However, any nanoclusters or nanoparticles formed would not be visible to the naked eye. This experimental setup aimed to discern the specific influence of the plasma process on the aluminum powder, thereby providing insights into the mechanism of nanoparticle deposition and surface alteration during the magnetron plasma treatment. Initially, the survey spectra (Figure 6a) and elemental composition (Table 1) measurements were performed for ball-milled Al powder, treated under magnetron plasma Al powder, which involves nanoparticle deposition, and separately synthesized Al nanoparticles on a quartz substrate. The results of ball-milled Al powder revealed the top layer elemental composition of C, O, Al, and a small amount of Mg additive with concentrations of 41.9 at.%, 33.8 at.%, 21.1 at.%, and 3.2 at.%, respectively. The Mg additives are acceptable because the primary material was taken as waste from an industry process with up to 4% Mg as an additive. The analysis of powder treated under magnetron plasma showed quite similar elemental composition, while the main difference was seen as a reduction in carbon and increment of oxygen. Carbon reduction is related to two processes: Plasma cleaning and Al nanoparticle deposition. The plasma treatment is commonly used for surface cleaning. Generally, Ar, N, or O gas are used for plasma surface cleaning to remove carbon-based contaminants from the atmosphere [47]. In our case, we used hydrogen gas, whose atoms transfer much less energy in plasma compared to Ar, N, or O gas atoms. However, it could still initiate a plasma cleaning procedure. Simultaneously, Al nanoparticles were deposited on ball-milled Al powder. Those freshly deposited Al nanoparticles relatively reduce the amount of C on top of the treated powder. In total, we observe a 28% carbon reduction compared to ball-milled powder. In the meantime, the analysis of separately synthesized Al nanoparticles on quartz substrate disclosed 15.8 at.% of C, 59.8 at.% of O, and 24.4 at.% of Al.

To understand the oxide-based chemical bonds, the O 1s peak was analyzed (Figure 6c). O 1s was normalized and compared for all three types of samples. It can be seen that all three peaks have similar shapes, while the one attributed to Al nanoparticles lacks the part on lower binding energies. The main part of the peaks can be attributed to the Al–O compound at a binding energy of about 531.5 eV. This was confirmed by analyzing Al 2p peaks for separate samples (Figure 6b,d,f). Meanwhile, the part at lower binding energies involves Mg–O chemical bonds, with binding energies at about 531.0 eV. This can be approved by elemental concentration results showing Mg existence at ball-milled Al powder and treated under magnetron plasma Al powder only. Normally, Mg tends to form oxide/hydroxide-based compounds. Another explanation of the “shoulder” existence at lower binding energies can be attributed to the presence of suboxides. When aluminum is ball-milled, the newly exposed surfaces might not completely oxidize to Al_2_O_3_, leading to the formation of various suboxides instead. These suboxides (like AlOₓ) have lower binding energies compared to fully oxidized Al_2_O_3_, creating a distinctive shoulder at lower binding energies in the spectrum. Similar tendencies were observed by other authors analyzing oxide-based compounds [64,65,66,67]. The Al 2p chemical bond analysis of Al nanoparticles (Figure 6b) disclosed the existence of the Al^3+^ component only at 74.2 eV binding energy, representing an Al_2_O_3_ chemical compound. This means that deposited Al nanoparticles were straightly oxidized, probably after material extraction from the vacuum chamber to the atmosphere. Meanwhile, the chemical bond fitting results of ball-milled powder (Figure 6f) showed Al^3+^ and Al^0^ phases at 74.2 eV and 71.3 eV, respectively, representing both oxide (Al_2_O_3_) and metal Al structures. It is important to mention that the Al^3+^ component covered 56.4 area %, while the Al^0^–43.6 area % of the total Al 2p peak, meaning that the oxide phase is dominant. Similarly, the Al powder treated under magnetron plasma disclosed the same phases, including Al^3+^ and Al^0^, while the main difference was that Al^3+^ covered 70.6 area % and Al^0^ covered 29.4 area %. These results showed the Al^0^/Al^3+^ ratio changed from 0.77 to 0.42 after ball-milled Al powder treatment under magnetron plasma, indicating the reduction of the Al metallic phase and the increment of Al_2_O_3_. We presume that this might be related to the oxidation of Al nanoparticles when Al powder was extracted into the air atmosphere after plasma treatment under a magnetron atmosphere, where Al nanoparticles were deposited. However, some previous articles showed that Al_2_O_3_ incorporation into Al powder could enhance hydrogen production via the Al powder reaction with water [68,69].

The plasma influence on the Al powder top layer was modeled by TRIM software (SRIM-2008.04), and the results are presented in Figure 6e. As discussed in the explanation of the XPS results, magnetron-initiated plasma could alter the surface by several nanometers. Our calculations showed that hydrogen plasma could penetrate up to 150 Å, while the ion range (the highest hydrogen ion concentration) was calculated at 39 Å. We presume that some of the hydrogen ions could stick at the top Al layer and positively influence H_2_ production via hydrolysis reactions as well.

Additionally, this study found no observable changes in the XRD patterns of ball-milled and plasma-treated aluminum. The aluminum peaks displayed consistent cubic crystal orientations of (111), (200), and (220), with nearly the same lattice parameters (a = 4.065 Å) for both samples. Hydrogen ions are very tiny; thus, any alterations they induce would likely affect only the surface or topmost layers. Consequently, such changes may not be observable via XRD, which primarily examines bulk material properties at a micrometer level.

### 3.3. SEM Surface Morphology Analysis

**Figure 7:**
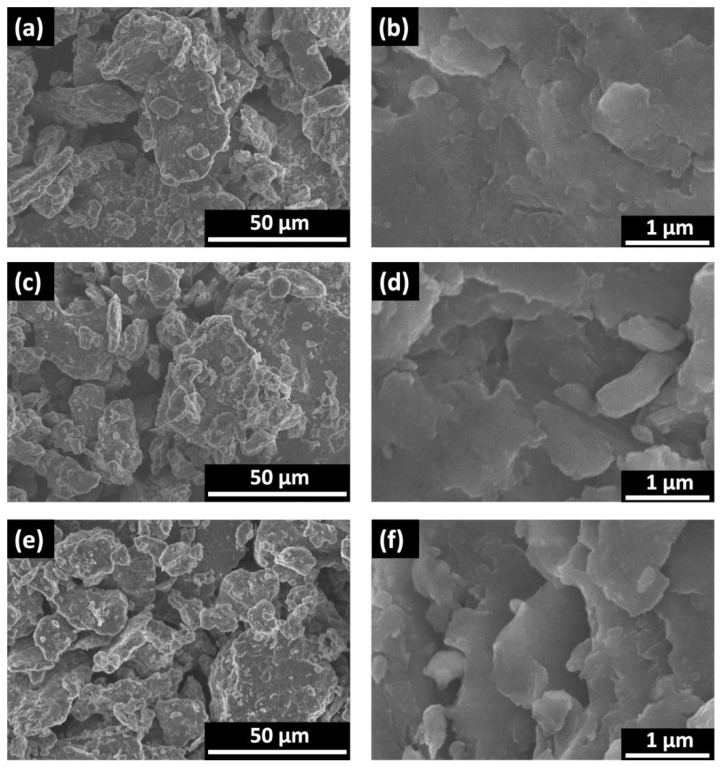
SEM views at different magnifications: (**a**,**b**)—ball-milled Al powder, (**c**,**d**) Al powder treated under diode plasma, (**e**,**f**)—Al powder treated under magnetron.

The morphology views of ball-milled Al powder are presented in Figure 7a. As it can be seen, the powder includes particles of various sizes and shapes, ranging from hundreds of nanometers to tens of micrometers, while the higher magnitude revealed a relatively flat powder structure with small slivers (Figure 7b). Additional examination revealed that there is no notable disparity in powder morphology when comparing large-scale images of both types of treated powder with untreated powder—both contain powder at both micro and nano levels (Figure 7c,d). Meanwhile, analysis at higher magnitudes disclosed negligible changes, showing the appearance of some randomly distributed small cracks or shape-edged structures. These alterations were more prominently observed when analyzing aluminum powder treated under magnetron-initiated plasma compared with diode plasma treatment due to: (i) Magnetron-initiated plasma is more intensive with better capability to clean powder surface contaminants and penetrate into the top layers by creating some cracks; (ii) magnetron treatment includes Al nanoparticle deposition, which could highlight some Al powder structures (Figure 7d,f). These alterations, including the appearance of small cracks or shape-edged structures, might have a positive influence on hydrolysis reaction kinetics.

### 3.4. Application to PEM Fuel Cell

**Figure 8:**
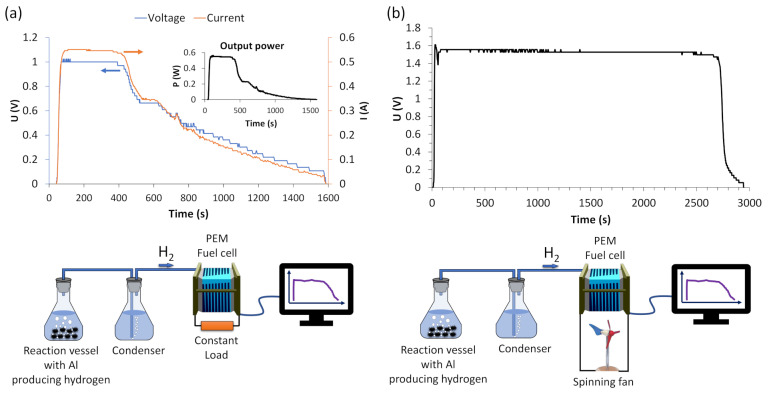
Fuel cell performance with (**a**) a constant load, and (**b**) a spinning fan.

In this study, we investigated the electricity generation capabilities of a proton exchange membrane (PEM) fuel cell utilizing 0.2 g of magnetron plasma-treated aluminum powder in conjunction with an alkaline solution (50 mL of 0.125 M). Specifically, we explored two scenarios: one with a constant load connected to the fuel cell (Figure 8a) and another with a spinning fan (Figure 8b). 

Figure 8a depicts the voltage and current generation over time during the hydrolysis process. Power generation commenced shortly after the reaction initiation, ensuring a consistent hydrogen flow. For over 300 s, the output power remained stable at around 0.55 W, with a voltage of 1 V and a current of 0.55 A. The increased hydrogen flow rate indicates a greater number of hydrogen molecules accessible for chemical reactions within the PEM fuel cell. Subsequently, as the hydrogen flow decreased, the voltage and current gradually diminished over time, eventually reaching zero after approximately 1500 s, when the reaction ceased entirely. Of course, a higher amount of aluminum would ensure sustained electricity generation.

In another scenario depicted in Figure 8b, a spinning fan was employed instead of a constant load connected to the fuel cell. In this setup, only voltage could be registered because the fan utilized is a brushless DC motor. Consequently, its resistance cannot be directly measured with a multimeter while in motion due to the continuously changing resistance. However, when the fan is not spinning, the resistance between its terminals measures approximately 33 ohms. Nonetheless, a steady voltage generation of 1.5 V was attained as the hydrolysis commenced and continued for approximately 2700 s. The prolonged duration compared to the constant load suggests that the constant load may consume more energy than the fan. On the other hand, a spinning fan could also generate electricity itself, potentially contributing to sustaining the prolonged electricity generation by the PEM fuel cell system.

## 4. Conclusions

In this study, the innovative application of low-temperature plasma treatment on aluminum to enhance hydrogen generation through the hydrolysis reaction was investigated. The experiments involved treating waste Al scraps with ball-milling and low-temperature plasma treatment using diode and magnetron-initiated plasma methods. The findings revealed that magnetron-initiated plasma treatment significantly enhanced the kinetics of the hydrolysis reaction, leading to quicker hydrogen generation. Specifically, magnetron plasma-treated aluminum powder produced 240 mL of H_2_ within 1325 s, compared to untreated powder, which achieved the same H_2_ generation in 2603 s using a 0.125 M solution. The calculated activation energy of 32.30 kJ mol^−1^ falls within the range reported by other authors, which typically ranges from 22.57 to 73.0 kJ mol^−1^. This similarity suggests enhanced reactivity with magnetron plasma treatment. 

Furthermore, the XPS analysis indicated that magnetron plasma treatment not only cleaned the powder surface but also deposited Al nanoclusters, contributing to enhanced hydrogen generation kinetics, even with relatively low solution molarity. Various chemical and physical processes, including adsorption, the formation and desorption of volatile compounds, and preferential sputtering of surface atoms, may have collectively contributed to the formation of an active altered layer in the near-surface region during plasma treatment. The XPS revealed that the Al^0^/Al^3+^ ratio decreased from 0.77 to 0.42 after treating ball-milled Al powder with magnetron plasma, indicating a reduction in metallic Al and an increase in Al_2_O_3_. This change may have resulted from the oxidation of Al nanoparticles during exposure to air after plasma treatment. It has been suggested by previous research that incorporating Al_2_O_3_ into Al powder can enhance hydrogen production when reacting with water.

Moreover, electricity generation experiments were conducted using a proton exchange membrane fuel cell with 0.2 g of magnetron plasma-treated aluminum powder. The results demonstrated that, using a constant load of 2.5 ohms, the voltage of 1 V and current of 0.55 A were stable for around 300 s. On the other hand, even this small amount of plasma-treated Al powder sustained stable electricity generation for almost 3000 s when utilized with an external load such as a spinning fan. These promising results highlight the potential of plasma treatment for enhancing H_2_ production via the hydrolysis reaction.

However, further investigations are needed to gain a deeper understanding of the underlying processes and to optimize the conditions for practical application of this technique. Future research could explore alternative plasma treatment methods and further refine process parameters to maximize the efficiency of hydrogen production from aluminum scraps. Overall, the study contributes to advancing sustainable hydrogen production methods and offers insights into the potential applications of plasma treatment in waste utilization and clean energy technologies.

## Figures and Tables

**Table 1:** Elemental composition by XPS.

Sample	Elemental Concentration, at. %			
	C	O	Al	Mg
Ball milled//@1	41.9	33.8	21.1	3.2
Treated under magnetron plasma//@2	30.1	43.5	24.4	2.0
Al nanoparticles//@3	15.8	59.8	24.4	-

## Data Availability

The original contributions presented in the study are included in the article, further inquiries can be directed to the corresponding author.
